# Biomarkers for cardiovascular risk prediction: from pioneering to evidence-based use in clinical practice

**DOI:** 10.1007/s12471-022-01737-0

**Published:** 2022-11-15

**Authors:** M. E. W. Hemels

**Affiliations:** 1 Department of Cardiology, Rijnstate Hospital, Arnhem, The Netherlands; 2 Department of Cardiology, Radboud University Medical Center, Nijmegen, The Netherlands

An ageing population has an increasingly higher risk of cardiovascular complications such as coronary heart disease, stroke, heart failure, peripheral arterial disease and/or aortic disease. Two-thirds of people aged > 60 years and more than 85% of people aged > 85 years have some form of cardiovascular disease (CVD) [1]. Between now and 2050, the number of people in Europe aged 75–84 years is estimated to increase by 60%. This substantial increase in cardiovascular morbidities will contribute to a rising burden on the healthcare system and highlights the urgent need for better risk assessment and risk prediction in order to prevent future disease and hospitalisations related to cardiovascular complications. Moreover, even though risk prediction tools are recommended in the European guidelines, they are often not adequately implemented in daily clinical practice [2].

In this issue of our journal, Zelis and colleagues studied the potential role of the biomarkers high-sensitivity cardiac troponin T (hs-TnT) and N‑terminal pro-B-type natriuretic peptide (NT-proBNP) in estimating the risk of major adverse cardiovascular events (MACE) for patients aged ≥ 65 years presenting at the emergency department (ED) with non-cardiac medical complaints [3]. In the studied population (*n* = 431, mean age 79 years, 47% females) the main reasons for the ED visit were infectious disease (29.5%) or disease of the digestive system (28.8%). More than half of these patients had at least one cardiovascular comorbidity (52.4%) or at least one cardiovascular risk factor (85.3%). Values of hs-cTnT and NT-proBNP were above reference range in 69.5% and 34.8% of patients respectively, and 60.4% of patients had an abnormal ECG. During a follow-up period of 1 year, one out of five patients (20%) developed a MACE, with a median time until MACE of 50 days (interquartile range 6 to 160 days). The most prevalent presentations of MACE were heart failure (52.3%) and stroke/transient ischaemic attack (26.7%), and the cardiovascular death rate was 4.9%. Both hs-cTnT and NT-proBNP measured at the index visit appeared to be predictive of subsequent MACE (area under the curve of 0.74 for both). In the multivariable analysis, when corrected for other cardiovascular risk factors, NT-proBNP was shown to be an independent predictor of MACE with a hazard ratio (HR) of 1.35 (95% confidence interval [CI] 1.09–1.67), while hs-cTnT was not (HR of 1.05, 95% CI 0.77–1.44).

This interesting study shows a high incidence of MACE in older patients in the first year after visiting the ED with non-cardiac medical complaints, and is the first prospective study evaluating the predictive ability of hs-cTnT and NT-proBNP in this specific population. In comparison, the incidence of MACE in adults aged ≥ 75 years undergoing non-cardiac surgery (NCS) is about 9.5%, and for these patients guidelines recently published by the European Society of Cardiology recommend a careful preoperative evaluation [1, 4]. In addition, the biomarkers hs-cTnT and NT-proBNP have also proven to be predictive of adverse outcomes in this NCS patient population, and are therefore included in perioperative risk estimation calculators/scores besides focused history, physical examination, and assessment of functional capacity. To be more specific: in patients with known CVD, cardiovascular risk factors (including age ≥ 65 years) or symptoms suggestive of CVD, it is recommended to measure hs-cTnT or hs-cTnI (Class I recommendation) before intermediate or high-risk NCS, and it should be considered to measure BNP or NT-proBNP (Class IIa recommendation) [1]. This argues for a better collaboration with anaesthetists.

Biomarker-based risk scores, such as the ABC-AF scores (age, biomarkers, clinical history) for stroke and bleeding risks, appear to show better discrimination in daily clinical practice than traditional risk scores in specific patient populations (e.g. ongoing trial: the Dutch-GERAF study, NCT05337202) [5]. However, we need to look critically at the added value of biomarker assessment in daily clinical practice. It should not lead to unnecessary diagnostic tests or unproven treatment measures. However, given the fact that the incidence of heart failure was the most common complication after the ED visit, it is tempting to speculate whether timely recognition followed by an early intervention in elderly patients with an elevated NT-proBNP could prevent the development of symptomatic heart failure after discharge [6]. This effect should be investigated in randomised studies. Furthermore, it is reassuring to learn that in patients without cardiac complaints presenting at the ED hs-cTn appeared not to be an independent predictor of MACE in the post-ED phase.

## In conclusion

In order to reduce the burden of cardiovascular disease in Europe, healthcare policymakers will need to allocate preventive measures and healthcare resources with proven benefit. Early disease recognition by opportunistic screening might also play an important role, in particular to the more vulnerable populations and to those with cardiovascular risk factors. Given the fact that reliable tools predicting cardiovascular outcomes in elderly patients visiting the ED are lacking, the study of Zelis et al. adds important new information and opens new doors to further studies on biomarker-based risk assessment. In future trials—with a multidisciplinary approach—the guiding role of specific biomarkers in both cardiovascular risk prediction and the effects of early interventions with or without telemonitoring should be studied, aiming to efficiently reduce cardiovascular disease burden.
